# Effects of a transitional care intervention on readmission among older medical inpatients: a quasi-experimental study

**DOI:** 10.1007/s41999-022-00730-5

**Published:** 2022-12-23

**Authors:** Lisa Fønss Rasmussen, Ishay Barat, Anders Hammerich Riis, Merete Gregersen, Louise Grode

**Affiliations:** 1 Department of Research, Horsens Regional Hospital, Horsens, Denmark; 2 Department of Medicine, Horsens Regional Hospital, Horsens, Denmark; 3 Enversion A/S, Aarhus, Denmark; 4 Department of Geriatrics, Aarhus University Hospital, Aarhus, Denmark

**Keywords:** Readmission, Mortality, Days alive and out of hospital (DAOH), Older medical patients, Transitional care interventions, Continuity of care

## Abstract

**Purpose:**

To evaluate the effect of a transitional care intervention (TCI) on readmission among older medical inpatients.

**Methods:**

This non-randomised quasi-experimental study was conducted at Horsens Regional Hospital in Denmark from 1 February 2017 to 31 December 2018. Inclusion criteria were patients ≥ 75 years old admitted for at least 48 h. First, patients were screened for eligibility. Then, the allocation to the intervention or control group was performed according to the municipality of residence. Patients living in three municipalities were offered the hospital-based intervention, and patients living in a fourth municipality were allocated to the control group. The intervention components were (1) discharge transportation with a home visit, (2) a post-discharge cross-sectorial video conference and (3) seven-day telephone consultation. The primary outcome was 30-day unplanned readmission. Secondary outcomes were 30- and 90-day mortality and days alive and out of hospital (DAOH).

**Results:**

The study included 1205 patients (intervention: *n* = 615; usual care: *n* = 590). In the intervention group, the median age was 84.3 years and 53.7% were females. In the control group, the median age was 84.9 years and 57.5% were females. The 30-day readmission rates were 20.8% in the intervention group and 20.2% in the control group. Adjusted relative risk was 1.00 (95% confidence interval: 0.80, 1.26; *p* = 0.99). No significant difference was found between the groups for the secondary outcomes.

**Conclusion:**

The TCI did not impact readmission, mortality or DAOH. Future research should conduct a pilot test, address intervention fidelity and consider real-world challenges.

**Trial registration:**

Clinical trial number: NCT04796701. Registration date: 24 February 2021.

**Supplementary Information:**

The online version contains supplementary material available at 10.1007/s41999-022-00730-5.

## Key summary points

**Aim:**

To evaluate the effect of a transitional care intervention among older medical inpatients.

**Findings:**

The follow-home transitional care intervention did not have an impact on readmission and mortality rates.

**Message:**

In a real-world context, several aspects make it difficult to measure the true impact of a multicomponent intervention.

**Supplementary Information:**

The online version contains supplementary material available at 10.1007/s41999-022-00730-5.

## Introduction

Transitions between hospital and primary care settings are identified as high-risk scenarios for patient safety that often result in adverse events, such as unplanned readmissions [1]. The share of unplanned hospital readmissions among somatic patients was 11.7% in Denmark in 2018 [2]. Approximately 20% of all hospitalised older medical patients are readmitted within 30 days after discharge [3]. Among other reasons, readmissions are caused by poor quality of care after discharge [4, 5], insufficient discharge planning or insufficient communication between hospital and home care or general practitioners (GPs) [6, 7].

Transitional care interventions (TCIs) are one strategy for preventing readmissions. Transitional care is defined as ‘a set of actions designed to ensure the coordination and continuity of healthcare as patients transfer between different locations or different levels of care within the same location. Representative locations include (but are not limited to) hospitals, subacute and post-acute nursing facilities, the patient’s home, primary and specialty care offices, and assisted living and long-term care facilities’ [8]. Over the past few decades, TCIs have been widely examined. TCIs are delivered by various healthcare professions, such as nurses, pharmacists, physiotherapists, GPs and/or geriatricians [9–12]. Some studies have focused on either the pre- or post-discharge periods, while others have focused on both. Other studies have targeted specific diagnoses or patient groups [10, 13–15]. The effects of TCIs on readmission rates vary. However, most studies have reported positive findings [16–19].

Despite the diversity of studies, we have yet to examine the effects on readmissions among an unselected medical patient group in a regional setting where the demographics, geographical distances and healthcare services differ from large urban settings.

The objective of this study was to examine the effect of a hospital-based TCI on readmissions among older medical patients admitted to a general medical ward at a regional hospital. We hypothesised that cross-sectorial collaboration with a follow-home function, post-discharge cross-sectorial video conferences and telephone consultations would reduce the number of all-cause unplanned readmissions compared to patients who received usual care without this cross-sectorial collaboration.

## Methods

### Design and setting

This study was a non-randomised quasi-experimental study conducted at Horsens Regional Hospital from 1 February 2017 to 31 December 2018. Horsens is a public hospital in the Central Denmark Region with 21,746 acute hospital admissions in 2017 and a total of 240 beds. The hospital receives patients primarily from the following four municipalities: Odder, Hedensted, Skanderborg and Horsens. The total number of inhabitants in 2017 was 218,286. Of these, 15,907 (7.3%) were older than 75 years, and of those, 57% were women. The share of inhabitants ≥ 75 years old was 7.1% in Horsens municipality, 6.4% in Skanderborg, 8.1% in Hedensted and 8.9% in Odder [20]. The Horsens Medical Department is divided into three subspecialised medical wards. However, the study was conducted in Medical Ward 2, a 27-bed ward with the following specialities: internal medicine, geriatric medicine and endocrinology.

### Population

Patients were eligible for inclusion if they met the following criteria: ≥ 75 years old, living in the municipalities of Odder, Skanderborg, Hedensted or Horsens and had been admitted to Medical Ward 2 for at least 48 h. Each weekday morning, patients were screened for inclusion criteria. If the patient was eligible for inclusion, the municipality of residence was determined. Then, allocation was conducted according to the patients’ municipality of residence.

### Intervention group

In addition to usual care, patients were offered the follow-home TCI if they lived in the municipalities of Hedensted, Odder or Skanderborg at the time of the index admission. All patients in the intervention group signed an informed consent form. Eligible patients were consecutively approached and enrolled.

### Control group

Patients living in the municipality of Horsens at the time of the index admission received the usual care. The control group was identified through the CROSS-TRACKS register [21] during the post-intervention analysis process. According to The Data Protection Act §10, informed consent was not needed to retrieve data from the register.

All relevant legal approvals to collect data on the intervention and control groups were obtained. For details, see Declarations.

The study is reported according to the ‘Guidelines for Reporting Non-Randomised Studies’ [22] and ‘Conducting and Reporting Trials for Older People’ [23].

### Follow-home intervention

‘The Template for Intervention Description and Replication (TIDieR) Checklist and Guide’ [24] has been used to ensure a transparent and thorough description of the intervention.

### Intervention development

The intervention was originally developed in an urban setting in Denmark [25]. It was adjusted to a regional context with different demographics, geography and budget by a hospital-based expert panel in 2016. The panel consisted of healthcare professionals from Horsens Regional Hospital: the head consultant, the head nurse, the nurse manager, registered nurses from the Medical Department and the project leader of Safer Patient Flow. The services should bridge and ensure continuity of care and treatment between sectors. This should be assured by close, comprehensive and timely communication with patients, relatives and healthcare collaborators.

### Delivering the intervention

Three healthcare professionals from Medical Ward 2 were dedicated as project workers. Two were registered nurses with four and 12 years of job experience, respectively, and one was a social and healthcare assistant with 17 years of experience.

### Intervention components

Overall, the hospital-based intervention consisted of three components covering the pre-discharge and post-discharge phases.

1. Pre-discharge phase: discharge transportation and home visit
  On the day of discharge, patients were physically escorted home from the hospital by a project worker, either in a project car or by bedridden-patient transportation, if they were physically or mentally incapable of sitting in a car. Whichever means of transportation, a physical home visit was conducted immediately after the patient’s return home or on the first weekday afterwards. During the visit, the project worker ensured that the patient felt as safe as possible, basic human needs were met, a medication reconciliation was conducted, the patient’s nutritional status was assessed, fall prevention efforts were initiated and challenges and concerns were discussed and resolved whenever possible. A video conference for the following working day was arranged during the visit.
2. Post-discharge phase: cross-sectional video conference and telephone consultations
  A video conference was conducted on the following working day. The conference aim was to bridge the gap between hospital and primary healthcare professionals by ensuring (1) a smooth transition with timely and sufficient communication; (2) that questions, barriers and worries were handled; and (3) continuity of care and treatment plans were arranged. The project worker participated from the hospital, while the patient, district nurse and relatives participated from the patient’s home. If a video conference was not possible, a telephone conference was held instead. To facilitate the conference, one computer or tablet was provided for the project worker, and one was provided for the district nurses in each of the three participating municipalities.
  Telephone consultations with the project workers or geriatricians were offered for up to seven days after discharge. Patients, relatives, district nurses and GPs were given a direct phone number that was answered by either a project worker or a nurse coordinator 24/7. Among other things, questions about hospital-based treatment and care, modifications of the treatment and care plans and disease deterioration could be discussed during these consultations.

### Usual care

The usual care was provided equally to all patients in the intervention and control group, thus all patients receive the same level of standard care. At Horsens Regional Hospital, planning of the discharge begins no later than the second day after hospital admission. Overall, the nurses are responsible for the daily assessments of functionality and needs and for care plans and digital communication with the home care staff and practical matters in relation to the discharge. On admission day, a medication reconciliation is conducted by a hospital doctor, and a nurse begins the nursing discharge status document and checklist.

A nursing care plan is started the day after admission and digitally sent to the home care staff as a preliminary plan. This plan includes information about the expected discharge date, functional ability, need for home healthcare services after discharge and coordination in relation to the discharge. Families or other caregivers are only automatically involved in the plan if the patients explicitly want them to be involved in complex discharge situations. During admission, nurses assess and act on the need for assistive technology after discharge and the need for post-discharge follow-up from outpatient clinics, GPs, home care services, district dieticians and specialised services for specific patient groups.

The nurse sends the updated nursing care plan to home healthcare before 12 p.m. the day before discharge. On the day of discharge, plans and lists are updated, the hospital doctor draws up a medication reconciliation, and prescriptions are written. If specialised care or treatment is needed, the nurse contacts the district nurse by phone as a supplement to the digital plan.

Finally, the hospital doctor writes a discharge letter and digitally send it to the patient’s GP and district nurse. After discharge, the GP and district nurse are responsible for treatment and care. Online Resource 1 illustrates the components of the intervention and usual care.

### Variables and data sources

#### Outcome measures

The primary outcome was the all-cause 30-day unplanned readmission rate. The secondary outcomes were (1) the all-cause mortality rate measured at 30 and 90 days after discharge and (2) days alive and out of hospital (DAOH) and without GP visits (DAOHGP).

Readmission was defined as any unplanned hospital admission within four hours and 30 days after discharge [26]. The readmission could not be caused by accidents, violence or suicidal attempts. No specific diagnostic relations were drawn between the index admission and readmission [27]. The outcome assessment was not blinded. This, however, was not a problem or potential bias since all outcome data were retrieved from a register.

### Data sources

Data were obtained from CROSS-TRACKS [21]. These data cover home care services, pre-hospital services, prescriptions, hospital services, laboratory tests, socioeconomics data and data from the National Health Insurance Service Register, the Civil Registration System and the National Patient Register.

### Statistical analysis

The power analysis was calculated based on readmission rates of 22% in the control group and 12% in the intervention group [28]. The analysis found that 694 patients should be included in the study (347 in each group) to achieve a 5% significance level and a power of 90%, allowing for a 10% dropout rate.

Data management and statistical analysis plans were conducted prior to the study.

We used chi-square tests to describe and compare the baseline characteristics. When observations were not normally distributed, Wilcoxon rank-sum or Kruskal–Wallis tests were used.

A binary regression model was used to compare the 30-day readmission risk, 30- and 90-day mortality rates, DAOH and DAOHGP between the two groups. The 30-day readmission analysis was performed based on the first readmission. The relative risk (RR) with a 95% confidence interval (CI) was calculated. The statistical models were adjusted for covariates with a statistically significant difference between the intervention and control groups at baseline.

In this study, selection bias may be present, as the intervention and control groups were identified differently. Furthermore, identifying patients not receiving the allocated treatment was only possible if they were in the intervention group, not if they were in the control group. To mitigate selection bias and ensure the highest level of homogeneity between the groups, the analysis was based on data from all patients who met the inclusion criteria—that is, we analysed the effect of the assignment to the intervention at baseline rather than the effect of adhering to the intervention.^1^

Furthermore, for the primary outcome, analyses were performed in the following sub-groups: age group, gender, housing, civil status, social status, length of stay, Charlson Comorbidity Index (CCI), home care after discharge from index admission, municipality, blood samples at discharge, body mass index, admission year, vital signs at discharge, polypharmacy (five or more daily medicines [29]), number of admissions within one year prior to the index admission and visits to the GP within one year prior to index admission.

The Aalen–Johansen estimator displayed the mortality-adjusted cumulative incidence of 30-day readmissions. Both 30 and 90-day mortality rates were displayed using the Kaplan–Meier survival estimator.

DAOH was calculated by subtracting the days spent in the hospital (in the period within 30 days after discharge from index admission) from 30. DAOHGP was calculated as above, but visits to the GP were also subtracted. DAOH and DAOHGP were analysed as binary variables with cut-offs at ≥ 23 days.

Difference-in-difference (before and after) analysis was calculated based on 30-day readmission rates in patients who met the study inclusion criteria in all four participating municipalities in the pre-intervention period from 2015 to 2016. These data were compared to the 30-day intervention readmission rates. The results are presented as risk difference with standard errors (SEs) and p-values between the groups for 30-day readmissions.

The CCI scores were calculated based on International Classification of Diseases-10 diagnostic codes of 19 conditions [30, 31] and reported in the following categories: no comorbidity (score = 0), low (score = 1), medium (score = 2) and high (score = 3 +).

STATA version 17 [32] was used to conduct the statistical analysis and to display Aalen–Johansen- and Kaplan–Meier estimators and the difference-in-difference analysis. Statistical assumptions were tested prior to the analysis.

### Ethics

Approval was obtained from the Danish Data Protection Agency (case no. 1-16-02-105-19). The CROSS-TRACKS case no. was 008-2. Approval by the Central Denmark Region Ethical Committee was not required. Patients in the intervention group could withdraw their consent at any time. The intervention did not harm any patients. All procedures in this study were performed in accordance with the 1964 Declaration of Helsinki and its later amendments.

### Patient and public involvement

Neither the patients nor the public were involved in planning or evaluating this study.

## Results

### Participants

**Fig. 1:**
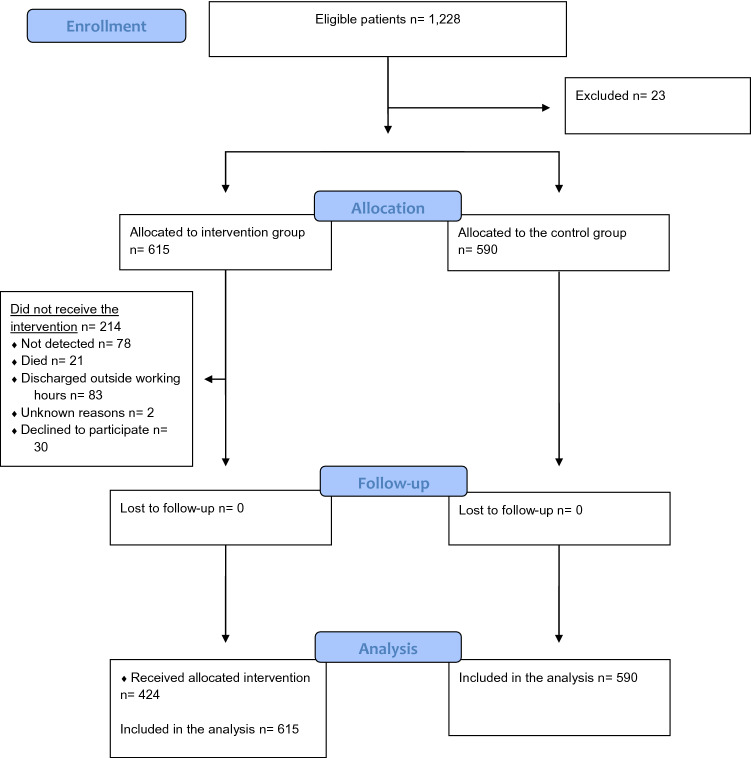
Flow diagram. Legend: an intention-to-treat analysis was conducted to address the potential bias that occurs when identification and inclusion/exclusion differ between the groups

During the study period, 1228 met the inclusion criteria, and 23 were excluded due to unknown reasons. Finally, 1205 patients were included in the study: 615 in the intervention group and 590 in the control group (Fig. 1). In the intervention group, 214 (17%) did not receive the intervention as planned. No patients were lost to follow-up, as we had CROSS-TRACKS data on all outcome variables.

The groups were similar in baseline characteristics, except for social status, type of home healthcare prior to index admission, and visits to the GP and out-of-hour doctor prior to the index admission. The median age was 84.3 years in the intervention group and 84.9 years in the control group. Of the intervention group, 53.7% were female and 46.3% were male, while 57.5% of the control group were female and 42.5% were male. See Table 1 for details.

**Table 1:** Baseline characteristics

	Intervention	Control	*p* value
Total, *n* (%)	615 (100)	590 (100)	
Gender, *n* (%)			0.185
Female	330 (53.7)	339 (57.5)	
Male	285 (46.3)	251 (42.5)	
Age, median (25–75 IQR)	84.3 (80.2–89.4)	84.9 (79.9–89.8)	0.599
Age groups, *n* (%)			0.514
75–79	146 (23.7)	150 (25.4)	
80–84	181 (29.4)	151 (25.6)	
85–89	150 (24.4)	148 (25.1)	
≥ 90	138 (22.5)	141 (23.9)	
LOS in days at index admission, median (25–75 IQR)	5.6 (3.8–7.6)	5.1 (3.7–7.1)	0.227
Housing, *n* (%)			0.156
Own home	548 (89.1)	540 (91.5)	
Nursing home	67 (10.9)	50 (8.5)	
Civil status			0.161
Married	222 (36.1)	178 (30.2)	
Divorced	64 (10.4)	64 (10.9)	
Not married	10 (1.6)	17 (2.9)	
Widow	305 (49.6)	314 (53.2)	
Unknown	14 (2.3)	17 (2.9)	
Social status, *n* (%)			0.045
Cohabiting	385 (62.6)	336 (57.0)	
Living alone	230 (37.4)	254 (43.0)	
CCI, *n* (%)			0.304
No comorbidity	180 (29.3)	146 (24.7)	
Low	109 (17.7)	118 (20.0)	
Moderate	106 (17.2)	112 (19.0)	
High	220 (35.8)	214 (36.3)	
Leading cause of index admission, *n* (%)			
Pneumonia	135 (22.0)	130 (22.0)	0.972
Cardiovascular disease	50 (8.1)	42 (7.1)	0.509
Other infections	79 (12.9)	82 (13.9)	0.591
Urinary tract infections	90 (14.6)	79 (13.4)	0.534
Gastrointestinal or constipation	12 (2.0)	15 (2.5)	0.488
Dehydration	30 (4.9)	26 (4.4)	0.698
Other endocrine/malnutrition	56 (9.1)	56 (9.5)	0.818
Dementia or delirium	16 (2.6)	14 (2.4)	0.799
Bone, muscle, connective tissue	29 (4.7)	25 (4.2)	0.688
Lesion or intoxication	23 (3.7)	25 (4.2)	0.659
Type for home healthcare before index admission, *n* (%)			
No service	187 (30.4)	119 (20.2)	0.000
Home visits by district nurse	297 (48.3)	380 (64.4)	0.000
Personal care	241 (39.2)	335 (56.8)	0.000
Practical help	276 (44.9)	363 (61.5)	0.000
Visits to GP within 30 days prior to index admission, *n* (%)			
Yes	430 (69.9)	369 (62.5)	0.007
Visits to out-of-hour doctor within 30 days of index admission, *n* (%)			
Yes	171 (27.8)	124 (21.0)	0.006
Number of drugs at discharge from index admission, median (25–75 IQR)	9 (6–12)	10 (7–12)	0.387
Polypharmacy, *n* (%)	552 (89.8)	529 (89.7)	0.957
Admissions 1 year prior to index admission, *n* (%)			0.989
Yes	296 (48.1)	285 (48.3)	0.952
BMI, *n* (%)	*N* = 519	*N* = 492	0.517
< 20	93 (17.9)	99 (20.1)	
20–25	200 (38.5)	188 (38.2)	
26–30	142 (27.4)	140 (28.5)	
> 30	84 (16.1)	65 (13.2)	

*CI* confidence interval, *RR* relative risk, *IQR* interquartile range, *LOS* length of stay, *CCI* Charlson comorbidity index, *BMI* body mass index

### Outcome data

All analyses were adjusted for the four covariates, with a statistically significant difference between the groups at baseline (see Table 1). The outcome estimates are reported in Table 2.

**Table 2:** Outcome estimates

	Intervention *n* (%)	Control *n* (%)	Crude estimate RR (95% CI)	Adjusted estimate RR (95% CI)
	N = 615	N = 590		
30-day readmission	128 (20.8)	119 (20.2)	RR: 1.03 (0.83,1.29) *p* = 0.782	RR: 1.00 (0.80, 1.26) *p* = 0.994
30-day mortality	115 (18.7)	108 (18.3)	RR: 1.02 (0.81, 1.29) *p* = 0.860	RR: 1.01 (0.80, 1.29) *p* = 0.919
90-day mortality	166 (27.0)	151 (25.6)	RR: 1.05 (0.87, 1.27) *p* = 0.582	RR: 1.07 (0.89, 1.30) *p* = 0.477
> 23 DAOH	464 (75.5)	453 (76.8)	RR: 0.98 (0.92, 1.05) *p* = 0.588	RR: 0.98 (0.92, 1.05) *p* = 0.593
> 23 DAOHGP	433 (70.4)	434 (73.6)	RR: 0.96 (95% CI: 0.89, 1.03) *p* = 0.223	RR: 0.96 (0.90, 1.03) *p* = 0.238

Adjusted for cohabiting, type of home healthcare prior to index admission, and visits to the GP and out-of-hour doctor prior to the index admission. *RR* relative risk, *CI* confidence interval, *DAOH* days alive and out of hospital, *DAOHGP* days alive and out of hospital and without GP visits.

### 30-day readmission

In total, 128 patients (20.8%) in the intervention group and 119 (20.2%) in the control group were readmitted within 30 days after discharge from index admission. No difference in the risk of readmissions was found between the groups in the adjusted analysis (RR: 1.00; 95% CI: 0.80, 1.26; *p* = 0.994).

The sub-group analysis showed a statistically significant difference between the intervention and control groups when looking at the sodium and potassium levels. Patients with abnormal sodium and potassium levels in the intervention group had a lower risk of readmission than patients with abnormal sodium and potassium levels in the control group (RR: 0.60, 95% CI: 0.37, 0.98, and RR: 0.59, 95% CI: 0.35, 1.00, respectively). For details on sub-group analyses, see Online Resource 2.

**Fig. 2:**
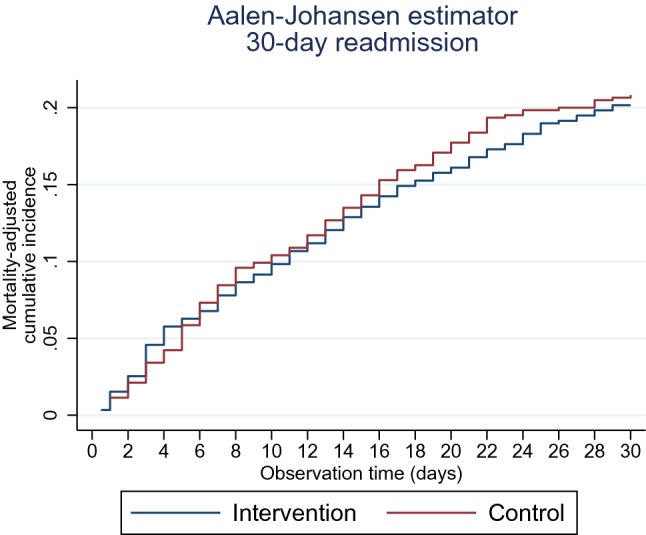
The mortality-adjusted cumulative incidence for 30-day readmission illustrated in an Aalen-Johansen plot. Legend: red line illustrates readmission among patients allocated to the control group, and blue line illustrates readmission in the intervention group

The mortality-adjusted cumulative incidence of 30-day readmission is displayed in Fig. 2.

### 30-day mortality

One hundred and fifteen (18.7%) patients from the intervention group and 108 (18.3%) from the control group died within 30 days after discharge. The adjusted risk for 30-day mortality was 1% higher in the intervention group than in the control group (RR: 1.01; 95% CI 0.80, 1.29; *p* = 0.919).

**Fig. 3:**
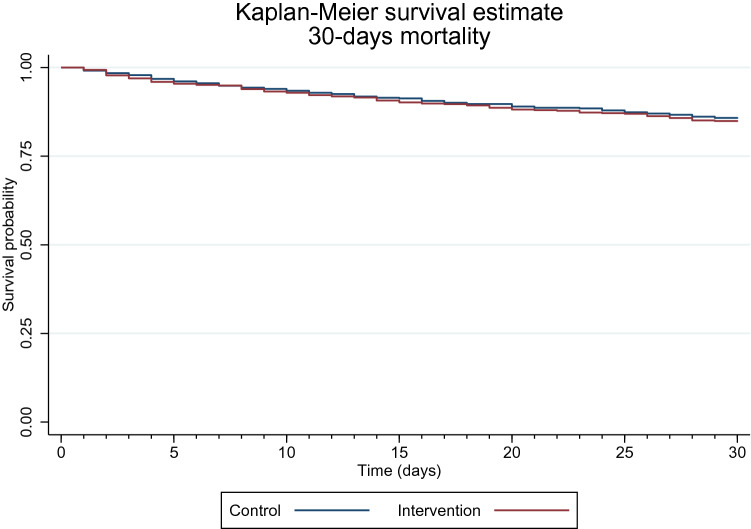
Kaplan–Meier survival estimates of 30-day mortality. Legend: red line illustrates mortality among patients allocated to the intervention group, and blue line illustrates mortality in the control group

The Kaplan–Meier survival estimates of 30-day mortality are displayed in Fig. 3.

### 90-day mortality

In total, 166 (27.0%) in the intervention group and 151 (25.6%) in the control group died within 90 days after discharge, resulting in a slightly higher adjusted risk of 7% (RR: 1.07; 95% CI 0.89, 1.30; *p* = 0.477).

**Fig. 4:**
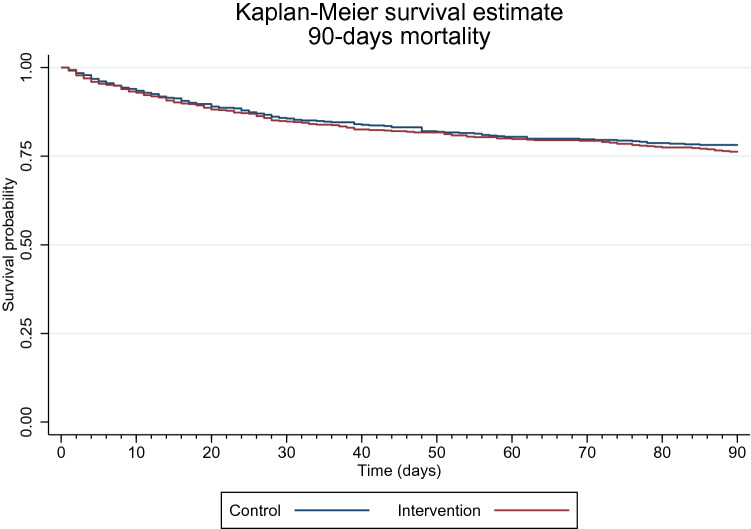
Kaplan–Meier survival estimates of 90-day mortality. Legend: red line illustrates mortality among patients allocated to the intervention group, and blue line illustrates mortality in the control group

The Kaplan–Meier survival estimates of 90-day mortality are displayed in Fig. 4.

### Days alive and out of hospital (DAOH)

In the intervention and control groups, 646 (75.5%) and 453 (76.8%) patients, respectively, were alive and out of hospital for more than 23 days within 30 days after discharge. This corresponds to a slightly lower adjusted risk of 2% in the intervention group compared with the control group (RR: 0.98; 95% CI 0.92, 1.05; *p* = 0.593).

### Days alive and out of hospital and GP visit (DAOHGP)

Almost similar numbers were seen when GP visits were taken into account. In other words, 433 (70.4%) of the patients in the intervention group were alive and out of hospital and had not visited the GP for more than 23 days within 30 days after discharge. The number was 434 (73.6%) for the patients in the control group. Thus, the adjusted risk was 4% lower among the intervention group compared with the control group (RR: 0.96; 95% CI 0.90, 1.03; *p* = 0.238).

### Other analysis

The risk of 30-day readmission two years before the intervention was 20.06% in the intervention group (Hedensted, Odder and Skanderborg) and 24.92% in the control group (Horsens). The risk after the intervention was 20.81% in the intervention group and 20.17% in the control group. Hence, the risk difference was + 0.75% (SE: 2.26%; *p* = 0.74) in the intervention group and − 4.75% (SE: 2.36%; *p* = 0.044) in the control group. The risk difference between the groups was 5.51% (SE: 3.27%; *p* = 0.092). The graphical display of the results can be found in Online Resource 3.

### Other results

No significant modifications were made to the intervention during the study period, and no adverse events were reported.

## Discussion

The intervention did not have a significant impact on 30-day readmission, 30- and 90-day mortality rates, DAOH or DAOHGP, as all RRs were close to 1. The no evidence of effect is generally caused by weakness in the design, implementation failure or no intervention effect. Plausible explanations to the lack of intervention impact in this study are discussed below.

### Comparison with previous research

Unfortunately, the results of no intervention impact on the outcome measures are common in the research field of TCIs. Over the last couple of decades, research has shown diverging effects on readmission and mortality among older medical patients. Some studies have reported positive impacts, while others have reported no or negative impacts [17, 18, 33–37]. Other studies found major implementation bottlenecks due to lack of resources, and information and organisational problems [38, 39]. This is in line with our experience with the TCI implementation. Most implementation barriers are related to information management, clinical uncertainties, sense of competences, perception of liability, expectations, standards of practice, financial disincentives and administrative constrains [40]. Some of these issues were, to different degrees, also present during our implementation.

### Design

Our intervention consisted of three components and was, thus, a low-intensity intervention.

A systematic review comparing the effects of different TCIs on readmissions among older medical patients found a noticeable impact among ‘high-intensity’ interventions and in interventions with duration of 1 month or more [17]. The low-intensity and short intervention duration may explain the lack of impact on the study outcomes.

Additionally, the intervention components were delivered merely by registered nurses and a social and healthcare assistant. Previous studies have documented that interventions with an interdisciplinary team positively impact readmission rates [28, 41]. If other healthcare professions had been involved, the intervention may have had a greater impact on the outcomes.

### Programme implementation

Some of the intervention components took a long time to implement in the municipalities. Most of the patients, relatives, district nurses and GPs in the three intervention municipalities did not use the seven-day telephone consultation offer in the first period of the study. The degree of usage and implementation could be assumed to have varied in and between municipalities. Additionally, the municipalities should have provided extra resources and time for the included patients to facilitate the post-discharge follow-up visits and video conferences. The video conference was a challenge for the district nurses and the involved patients. Technical difficulties, along with audio and visual challenges faced by patients, may have reduced the effect of the conference. In addition, scheduling conferences was difficult due to the high workloads among district nurses. Hence, some of the video conferences and physical visits were cancelled.

The GPs and district nurses were not thoroughly informed and instructed about the seven-day consultation offer’s purpose, benefit and usage. Thus, it was not fully implemented. This resulted in uncertainty about the post-discharge treatment responsibility among district nurses and GPs, and the consultation offer was not used as often as anticipated. This may have resulted in readmissions where it could have been prevented by communication between the GP, district nurse and the geriatrician.

Unfortunately, intervention fidelity was impossible to assess due to missing data entry. Therefore, we do not know the degree to which the intervention components were delivered as intended. If infidelity was present, it may have resulted in non-significant findings that were not due to the study design but rather to the intervention delivery [42].

The Medical Research Council’s [43] gold standard for developing a complex intervention was not applied when the intervention was adapted and modified. This could have resulted in critical aspects not being addressed. Among others, the council recommends assessing the feasibility prior to implementation to prevent problems of acceptability, compliance and delivery of the intervention [43]. If the gold standard had been followed, the abovementioned implementation challenges could have been reduced or prevented, resulting in a positive impact on outcome measures [44].

### Concurrent municipality-based projects and home care services

The initiative ‘Get Home Safe’ in Horsens home care was implemented during the study period. The aims were to (1) ensure the feeling of safe transitions from the hospital to patients’ own homes or community beds and (2) prevent hospital (re)admissions. As Horsens municipality served as our study’s control group, this initiative may have reduced the number of readmissions in the control group, thus resulting in a smaller study impact. In the same period, Odder home care also had an increased focus on improving the quality of transitions between the hospital and the patients’ own homes or community beds. Finally, Hedensted home care also implemented two intermediate beds in a nursing home during the period. These beds were used as an alternative to (re)admissions. Even though the readmission rates in the intervention group seemed constant over the two years, we must consider the possibility that these projects had an impact on the study outcomes.

Additionally, the healthcare services offered differ among the municipalities. For instance, acute care teams are the first to visit discharged patients in one municipality, while a social and healthcare helper or a social and healthcare assistant conducts the first visits in another municipality. One could speculate that these differences in knowledge and professional competencies may have affected the outcome estimates.

### Study population

The inclusion criteria in this study were few and broad, resulting in a rather unselected and heterogeneous population. Thus, the group comprised patients representing various of conditions, diseases and severities. This unselected patient group could lead to a null effect. A systematic review found a pronounced positive intervention impact among patients categorised as ‘high risk’ compared to unselected patients [17].

Older medical patients are a complex population characterised by severe diseases, comorbidity, polypharmacy, impaired nutritional status, reduced physical or mental functionality and limited ability to care for oneself [3]. In addition, this population has the highest utility of healthcare services compared to the general population [45]. One may argue that it is difficult to target and address the diversity of challenges and needs to prevent readmissions in this patient group.

The sub-group analysis found a weak statistical significant association between abnormal sodium and potassium levels and the risk of 30-day readmission. However, we would need further research to explain a possible causal relationship.

It is also important to remember that not all readmissions are preventable, as they are an expected part of a patient trajectory in this ageing patient group. It has previously been estimated that 23–36% of all readmissions are preventable, depending on the timespan between discharge and readmission [46, 47].

### Readmission and other outcomes

The difference in readmission rates between the groups was smaller after the intervention than before. The decreased risk difference over time was attributed to the control group. The rate decreased in the control group, while it was constant over time in the intervention group. Therefore, it seems that the intervention did not impact readmissions over time. This analysis suggests that a time trend did not affect the intervention effect.

To our knowledge, DAOH has not previously been used to analyse TCI impacts in this specific population. The outcomes have primarily been used to evaluate the effects in patients with heart conditions [48–51]. Hence, directly comparing our results with previous studies is difficult.

### Strengths and limitations

Our study has several strengths. Using CROSS-TRACKS’s wide range of data is a major strength. This enables detailed descriptions of the study population and a wide variety of sub-group analyses.

The heterogeneous population of general medical patients reflects a real-world population in regional hospital settings. This is also a strength, as most regional hospitals have general medical wards with a wide variety of medical conditions. This underlines the external validity. Compared to other similar studies, the sample size is rather large, which gives the study high power. Furthermore, the long study period prevented seasonal variation among the outcome measures, contributing to more valid results. Detection bias is not present in this study, as outcomes were identified through the CROSS-TRACKS register and assessed in all patients. Moreover, this study reports on DAOH and DAOHGP, which are upcoming outcomes within this research field. This adds a new perspective on the impact of a TCI.

Nevertheless, the study also has several limitations. First, the absence of frailty status and, thus, frailty sub-group analysis make it difficult to assess the intervention effect in this group. However, it would be impossible to screen the participants in the control group with a frailty tool, as they were identified through a register. However, we performed several sub-group analyses with variables included in different frailty tools (e.g. CCI, cohabiting and home healthcare prior to admission). Our sub-group analysis did not find any difference in the effect among the sub-groups.

Second, potential selection bias may threaten the internal validity, as the allocation was conducted according to the municipality of residence. In addition, the allocation process was untraditional, as one group was identified during hospitalisation and the other through a data register, thus preventing it from being a contemporary control group. This concern and possible attrition bias were handled in the analysis. The data were analysed based on the allocated intervention rather than the received intervention. Third, we conducted a difference-in-difference analysis to address the potential effect on readmission over a long study period. This showed no effect of time on readmissions in the intervention group. Fourth, performance bias may be present, as the healthcare services offered and provided were heterogeneous across the four municipalities. Fifth, the intervention fidelity was not calculated, thus making it difficult to assess whether the lack of intervention impact was affected by implementation failure. Hence, as with many similar studies [17], we could not identify essential components from unimportant ones. In addition, we did not conduct a process evaluation, which is often requested in these kinds of studies [52]. Sixth, the applicability of the results to other healthcare institutions should be carefully considered. The context, healthcare services, the financial payment schemes and patient characteristics may differ significantly nationally versus internationally. Sixth, the study’s expenses related to tablets, computers and a project-specific car are practical limitations to its applicability.

Lastly, we adjusted for covariates that were statistically significantly different between the groups in the statistical models. However, we did not have access to data on functional disability, socioeconomic status, prior falls and method of referral, which are known risk factors. This may have had a small impact on the outcome estimates.

### Clinical and research implications

This study illustrates the ‘real-world’ challenges in planning, implementing and evaluating a TCI in an ever-changing setting, with a complex study population and a long study period. To address this, it is essential to plan the study thoroughly based on previous research findings and to conduct pilot tests prior to implementation to ensure feasibility. Designing an interdisciplinary intervention is also essential to achieve a greater impact. In addition, future research should register implementation fidelity to provide a clear picture of whether the effects are caused by implementation failure or success, the study design or are solely due to the intervention. Future research should also address the importance of the implementation context and consider conducting a process evaluation. Finally, we recommended to screen participants for frailty to assess the intervention effect in this patient group.

## Conclusion

This modified TCI consisting of a discharge transportation, home visit, cross-sectorial video conference and seven-day telephone showed no evidence of effect. We do not know whether this was caused by implementation failure, weakness in the study design, lack of intervention effect or uncontrollable factors in the primary healthcare. The real-life implementation of this TCI was challenging and it seem crucial to be adherent the Medical Research Council’s recommendations and to evaluate the delivery of intervention components to assess the true effect of TCIs.

## Supplementary Information

Below is the link to the electronic supplementary material.

Supplementary file1 (PDF 130 KB)

Supplementary file2 (PDF 165 KB)

Supplementary file3 (PDF 52 KB)

## Data Availability

All data relevant to the study are included in the article or uploaded as supplementary information.

## Footnotes

1 This method of analysis was supported by the statistician Morten Frydenberg, who is one of the authors of the book *An Introduction to Stata for Health Researchers,* 5th Edition, Stata Press, 2022.
