# Supplementary material for: Effects of a transitional care intervention on readmission among older medical inpatients: a quasi-experimental study
Source: Eur Geriatr Med. 2022 Dec 23;14(1):131–44. doi: 10.1007/s41999-022-00730-5 (PMC9902414; doi:10.1007/s41999-022-00730-5)
Supplement: Supplementary file 1 — Supplementary file1 (PDF 130 KB) [file 41999_2022_730_MOESM1_ESM.pdf]

Online Resource 1
Intervention- and usual care components

Article title: Effects of a transitional care intervention on readmission among older medical inpatients: A quasi-randomised controlled trail

		Components	Usual care	Intervention
Pre-discharge	Admission day	Medication reconciliation by a medical doctor	X	X
		Start a nursing discharge status document	X	X
		Start a nursing discharge check list	X	X
	The day after admission	Assess the expected admission course	X	X
		Assess the functional ability	X	X
		Start the nursing care plan and send it to the home health care as a preliminary plan.	X	X
		Involve relatives in the discharge process if the patient wishes it	X	X
	During admission	Assess the need for assistive technology after discharge	X	X
		Regularly update the nursing care plan, the nursing discharge status document and nursing discharge check list	X	X
		Assess the need for post-discharge follow-up	X	X
		Send an updated nursing care plan to the home health care	X	X
	Discharge day	Ensure that the medication list is up-dated	X	X
		Medication reconciliation is done by a medical doctor	X	X
		Ensure that relevant prescriptions are made	X	X
		Offer a packed lunch to the patient	X	X
		Check the discharge check list	X	X
		Ensure the home transportation is planned and book a transport if needed	X	X

Online Resource 1
Intervention- and usual care components

Article title: Effects of a transitional care intervention on readmission among older medical inpatients: A quasi-randomised controlled trail

		Physically escort the patient home if the patient is physically capable		X
		Send a nursing discharge report to the home health care no later than 2 hours after discharge. This include a description of the admission course, status on nursing needs, functional ability, and coordination agreements with community-based nurse	X	X
		A medical discharge summary is send to the general practitioner	X	X
Post-discharge	The day of discharge	A home visit		X
	The day after discharge	A cross-sectorial video conference		X
	Up to 7 days after discharge	Telephone consultations was offered		X

Online Resource 1
Intervention- and usual care components

Article title: Effects of a transitional care intervention on readmission among older medical inpatients: A quasi-randomised controlled trail

Corresponding author:
Lisa Fønss Rasmussen
Department of Research
Sundvej 30
8700 Horsens
Denmark
E-mail: lirasm@rm.dk and lisafoenss@gmail.com
ORCID: 0000-0001-9405-9158

Co-authors:
Ishay Barat

Anders Hammerich Riis
ORCID: 0000-0002-6684-4068

Merete Gregersen
ORCID: 0000-0002-5365-7335

Louise Grode
ORCID: 0000-0003-0948-2328
