# Supplementary material for: Effects of a transitional care intervention on readmission among older medical inpatients: a quasi-experimental study
Source: Eur Geriatr Med. 2022 Dec 23;14(1):131–44. doi: 10.1007/s41999-022-00730-5 (PMC9902414; doi:10.1007/s41999-022-00730-5)
Supplement: Supplementary file 2 — Supplementary file2 (PDF 165 KB) [file 41999_2022_730_MOESM2_ESM.pdf]

Online Resource 2
Sub-group analysis for 30-day readmission

Article title: Effects of a transitional care intervention on readmission among older medical inpatients: A quasi-randomised controlled trail

Sub-groups		30-day readmission RR, CI 95%
		Overall adjusted RR: 1.00 (0.80;1.26)
		Adjusted RR for the intervention group compared with the control group
Age groups	75-79<br>80-84<br>85-89<br>≥ 90	1.19 (0.77;1.85)<br>0.75 (0.50;1.18)<br>1.32 (0.83;2.10)<br>0.90 (0.55;1.49)
Gender	Female<br>Male	1.03 (0.76;1.41)<br>0.97 (0.70;1.34)
Housing	Own home<br>Nursing home	1.01 (0.79;1.27)<br>0.98 (0.45;2.14)
Civil Status	Married<br>Divorced<br>Widow	0.96 (0.65;1.40)<br>1.12 (0.56;2.23)<br>0.96 (0.70;1.32)
Social status	Living alone<br>Co-habiting	0.87 (0.62;1.23)<br>1.12 (0.82;1.51)
LOS in days	≤ 3<br>3.1-6<br>6.1-9<br>9.1-12<br>≥ 12.1	1.47 (0.73;2.96)<br>0.87 (0.64;1.18)<br>1.33 (0.79;2.23)<br>0.64 (0.32;1.27)<br>1.30 (0.52;3.26)
CCI - comorbidity	No<br>Low<br>Moderate<br>High	1.09 (0.66;1.78)<br>0.79 (0.47;1.33)<br>1.47 (0.83;2.59)<br>0.94 (0.68;1.32)
Home healthcare services[A]	No service<br>Home visits by district nurse<br>Personal care<br>Practical help	1.18 (0.75;1.84)<br>0.91 (0.67;1.23)<br>1.18 (0.54;2.57)<br>1.00 (0.40;2.52)
Municipality	Skanderborg<br>Odder<br>Hedensted	1.07 (0.80;1.43)<br>1.28 (0.89;1.85)<br>0.84 (0.61;1.16)
Blood samples	CRP<br>Normal<br>Abnormal<br>eGRF<br>Normal<br>Abnormal<br>Hemoglobin<br>Normal<br>Abnormal<br>Sodium<br>Normal<br>Abnormal<br>Leucocytter<br>Normal<br>Abnormal<br>Potassium<br>Normal	 1.00 (0.55;1.81)<br>1.00 (0.78;1.28)<br><br>0.85 (0.61;1.17)<br>1.17 (0.85;1.61)<br><br>1.20 (0.80;1.82)<br>0.93 (0.71;1.21)<br><br>1.16 (0.90;1.50)<br>0.60 (0.37;0.98)*<br><br>1.14 (0.87;1.49)<br>0.75 (0.50;1.13)<br><br>1.14 (0.88;1.46)

Online Resource 2
Sub-group analysis for 30-day readmission

Article title: Effects of a transitional care intervention on readmission among older medical inpatients: A quasi-randomised controlled trail

	Abnormal	0.59 (0.35;1.00)*
	Albumin	
	Normal	1.40 (0.99;2.00)
	Abnormal	0.78 (0.58;1.05)
Admission year	2017	0.97 (0.70;1.34)
	2018	1.02 (0.75;1.41)
BMI	<20	0.62 (0.35;1.11)
	20-25	1.18 (0.82;1.70)
	26-30	0.83 (0.54;1.27)
	>30	1.83 (0.85;3.93)
Vital signs at discharge from IA	Systolic blood pressure	
	Normal	0.98 (0.78;1.24)
	Abnormal	1.82 (0.44;7.63)
	Pulse rate	
	Normal	0.97 (0.75;1.25)
	Abnormal	1.16 (0.71;1.90)
	Saturation	
	Normal	1.01 (0.79;1.27)
	Abnormal	0.98 (0.46;2.13)
	Respiratory rate	
	Normal	1.10 (0.80;1.51)
	Abnormal	0.91 (0.67;1.26)
Polypharmacy	No	1.29 (0.56;2.99)
	Yes	0.98 (0.77;1.24)
Admissions within 1 year prior to the IA	No	1.06 (0.75;1.50)
	Yes	0.96 (0.72;1.29)
Visits to the GP within 1 month prior to the IA	No	1.06 (0.70;1.63)
	Yes	0.98 (0.75;1.27)
Visits to the out-of-hour doctor within 1 month prior to the IA	No	1.12 (0.86;1.45)
	Yes	0.76 (0.50;1.15)
Time of discharge from the IA	Day shift	0.99 (0.78;1.27)
	Evening shift	0.97 (0.54;1.73)
	Weekday	0.99 (0.78;1.25)
	Weekend/holiday	1.21 (0.58;2.55)

LOS: length of stay; CCI: Charlson Comorbidity Index BMI: IA: index admission, GP

A: Rehabilitation is deleted due to few observations

* statistical significant

Corresponding author:
Lisa Fønss Rasmussen
Department of Research
Sundvej 30
8700 Horsens

Online Resource 2
Sub-group analysis for 30-day readmission

Article title: Effects of a transitional care intervention on readmission among older medical inpatients: A quasi-randomised controlled trail

Denmark
E-mail: lirasm@rm.dk and lisafoenss@gmail.com
ORCID: 0000-0001-9405-9158

Co-authors:
Ishay Barat

Anders Hammerich Riis
ORCID: 0000-0002-6684-4068

Merete Gregersen
ORCID: 0000-0002-5365-7335

Louise Grode
ORCID: 0000-0003-0948-2328
