# Supplementary figures and images for: Effects of a transitional care intervention on readmission among older medical inpatients: a quasi-experimental study
Source: Eur Geriatr Med. 2022 Dec 23;14(1):131–44. doi: 10.1007/s41999-022-00730-5 (PMC9902414; doi:10.1007/s41999-022-00730-5)

## Difference-in-difference

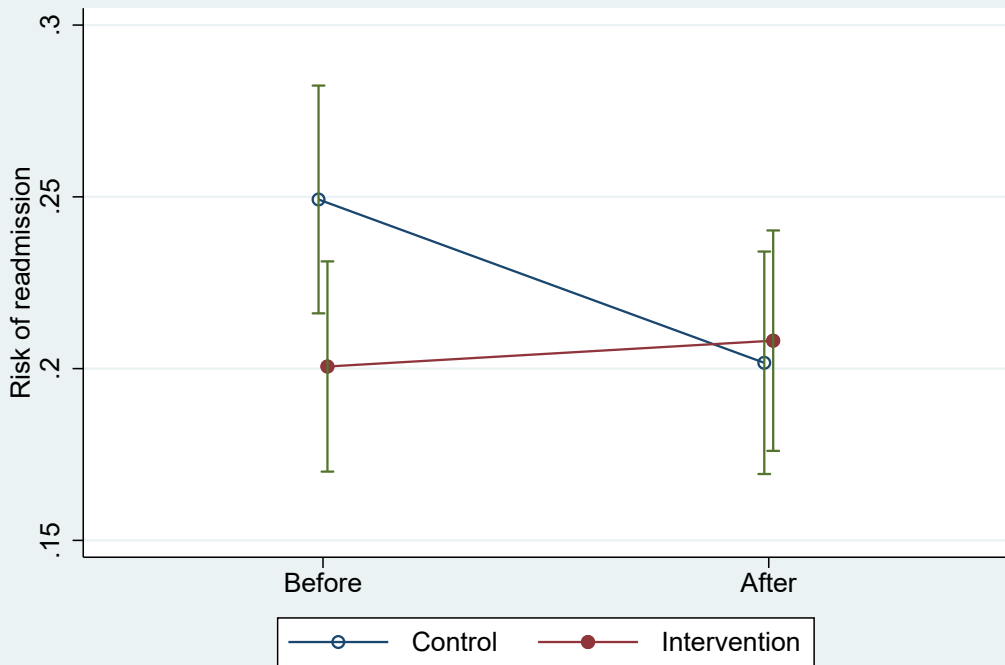

Supplement: Supplementary file 3 — Supplementary file3 (PDF 52 KB) [file 41999_2022_730_MOESM3_ESM.pdf]
